# Co-culturing experiments reveal the uptake of myo-inositol phosphate synthase (EC 5.5.1.4) in an inositol auxotroph of *Saccharomyces cerevisiae*

**DOI:** 10.1186/s12934-021-01610-6

**Published:** 2021-07-19

**Authors:** Erika Steele, Hana D. Alebous, Macy Vickers, Mary E. Harris, Margaret D. Johnson

**Affiliations:** 1 The University of Alabama, The Institute of Social Science Research, PO Box 8702161, Tuscaloosa, AL 35487 USA; 2 Department of Biological Sciences, School of Science, The University of Jordan, PO Box 11942, Amman-Jordan, Jordan; 3 Department of Biological Sciences, The University of Alabama, PO Box 870344, Tuscaloosa, AL 35487 USA

**Keywords:** Inositol auxotroph, *Saccharomyces cerevisiae*, Myo-inositol Phosphate Synthase (MIP), Protein secretion, Cellular supernatant, Extracellular vesicle, Co-culturing

## Abstract

**Background:**

Myo-Inositol Phosphate Synthase (MIP) catalyzes the conversion of glucose 6- phosphate into inositol phosphate, an essential nutrient and cell signaling molecule. Data obtained, first in bovine brain and later in plants, established MIP expression in organelles and in extracellular environments. A physiological role for secreted MIP has remained elusive since its first detection in intercellular space. To provide further insight into the role of MIP in intercellular milieus, we tested the hypothesis that MIP may function as a growth factor, synthesizing inositol phosphate in intercellular locations requiring, but lacking ability to produce or transport adequate quantities of the cell–cell communicator. This idea was experimentally challenged, utilizing a *Saccharomyces cerevisiae* inositol auxotroph with no MIP enzyme, permeable membranes with a 0.4 µm pore size, and cellular supernatants as external sources of inositol isolated from *S. cerevisiae* cells containing either wild-type enzyme (Wt-MIP), no MIP enzyme, auxotroph (Aux), or a green fluorescent protein (GFP) tagged reporter enzyme (MIP- GFP) in co- culturing experiments.

**Results:**

Resulting cell densities and microscopic studies with corroborating biochemical and molecular analyses, documented sustained growth of Aux cells in cellular supernatant, concomitant with the uptakeof MIP, detected as MIP-GFP reporter enzyme. These findings revealed previously unknown functions, suggesting that the enzyme can: (1) move into and out of intercellular space, (2) traverse cell walls, and (3) act as a growth factor to promote cellular proliferation of an inositol requiring cell.

**Conclusions:**

Co-culturing experiments, designed to test a probable function for MIP secreted in extracellular vesicles, uncovered previously unknown functions for the enzyme and advanced current knowledge concerning spatial control of inositol phosphate biosynthesis. Most importantly, resulting data identified an extracellular vesicle (a non-viral vector) that is capable of synthesizing and transporting inositol phosphate, a biological activity that can be used to enhance specificity of current inositol phosphate therapeutics.

## Background

Inositol phosphate, the product of MIP catalysis, and its phosphorylated derivatives are ubiquitous in all kingdoms of life and play vital roles in the spatial organization of central signaling pathways, including membrane trafficking, stress response, autophagy, neurodegenerative disorders, and polycystic ovary syndrome [1–9]. Inositol phosphate, the immediate precursor of free inositol, is generated from a tightly coupled oxidation and reduction reaction that requires NAD^+^ to proceed [6]. Proposed intermediates of this reaction, 5- ketoglucose 6-phosphate and inose, 2,1-phosphate, have not been isolated; it is believed that they remain tightly bound to the enzyme [6]. Due to the presence of MIP in a diverse array of prokaryotic and eukaryotic organisms, it is speculated that inositol phosphate biosynthesis arose early during evolution [10, 11]. MIP is the only enzyme known to catalyze the conversion of glucose-6 phosphate into inositol phosphate [6, 12].

Early experiments, utilizing bovine brain and plants (*Arabidopsis thaliana, Phaseolus vulgaris*)*,* and model eukaryote, *S. cerevisiae,* indicated that MIP associates with membranes and is secreted [13–17]. The discovery of MIP in yeast extracellular vesicles supports present and past research results concerning MIP location in intercellular space and organelles [15–22].

Importantly, the detection of MIP in extracellular vesicles established a basis for questioning the function of MIP in vesicles and for testing the hypothesis that MIP may function as a growth factor.

Extracellular vesicles have emerged as important mediators of intra- and intercellular communication [23–29]. They have been found to regulate a varied range of biological processes, and their pathophysiological roles are recognized in diseases such as neurodegenerative disorders, diabetes, and cancer [30–32]. Furthermore, extracellular vesicles have been detected in cerebrospinal fluid, an intermediary between blood and brain, in addition to other fluids, including saliva and seminal fluids [31]. Numerous investigations have shown that extracellular vesicles can be taken up and their internal contents act as signals [33–37].

Moreover, it has been shown that fungal cells produce extracellular vesicles, which share morphological and biochemical similarities with mammalian exosomes [17, 33], including an ability to modulate the function of immune cells [33]. Plant cells also produce exosome-like vesicles [38]**,** supporting the notion that vesicular release is a mechanism of trans-cell wall passage shared by cell-wall containing eukaryotes [38]. Thus, defining a function for MIP in extracellular vesicles broadens knowledge of the spatial control of inositol phosphate biosynthesis and enhances possibilities for controlling inositol phosphate metabolism in health and in disease.

Co-culturing experiments have been widely used to study cellular functions, such as transport, absorption, secretion, and cell-cell communication, since the pioneering, transfilter, metanephric induction studies of Grobstein [39]. Therefore, we employed an updated version of this technique to assess the ability of MIP to function as a growth factor [40, 41].

Supernatant from yeast cells containing either Wt-MIP enzyme (positive control), no MIP enzyme, Aux, (negative control), or MIP-GFP (enzyme tagged with GFP for detection) was employed to assess growth of an inositol auxotroph, Aux, in a cellular environment, which required external inositol. Results of transwell co- culturing experiments, designed to test these three different cellular supernatants for their ability to provide a source of inositol and sustain growth of an inositol requiring cell, are reported here.

## Results

### Validating the use of a GFP tagged MIP enzyme in co-culturing experiments

**Fig. 1:**
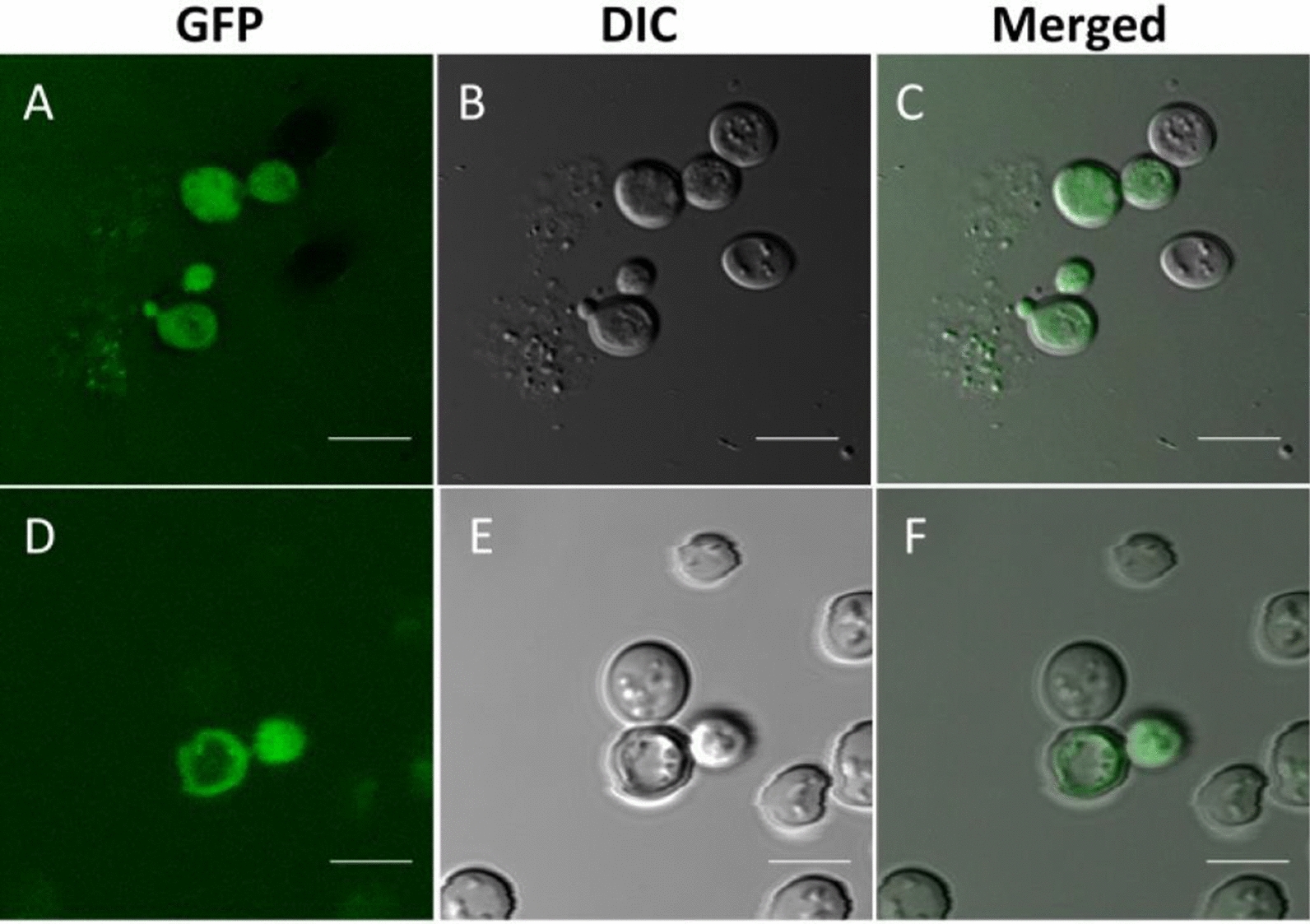
Confocal micrographs document MIP-GFP expression in *S. cerevisiae.* Confocal micrographs of MIP-GFP localization studies showed that MIP-GFP fluorescence is associated with internal membranes, cell walls, and the extracellular environment of *S. cerevisiae* AD. Corresponding DIC images, BE, were merged with fluorescent images AD, to better detail cellular structures, CF. Cells were visualized at ×400 magnification, bar represents 4 µm

**Fig. 2:**
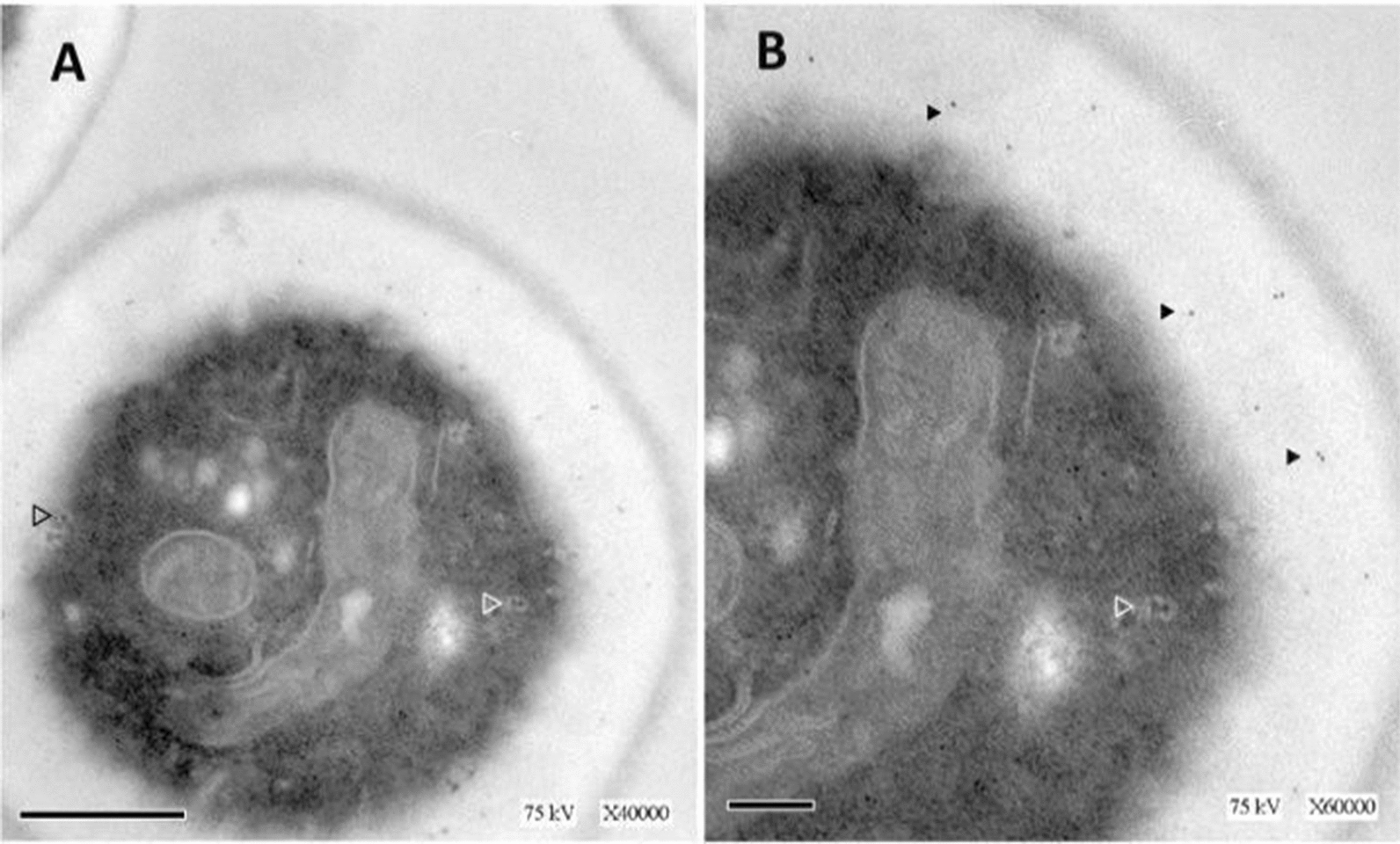
TEM studies detect Wt-MIP expression in extracellular vesicles of *S. cerevisiae.* TEM photomicrographs of MIP expression in Wt-MIP identified internal membranes, cell walls, and vesicles as sites of MIP expression **A**, **B**. Vesicles transporting MIP are associated with internal membranes (open arrows) and with the cell wall (closed arrows). Bar denotes 5 µm, **A**, and a magnification of ×40,000. In **B**, the bar represents 2 µm and a magnification of ×60,000. Cells were viewed at two different magnifications to better capture MIP localization and transport in vesicles

**Fig. 3:**
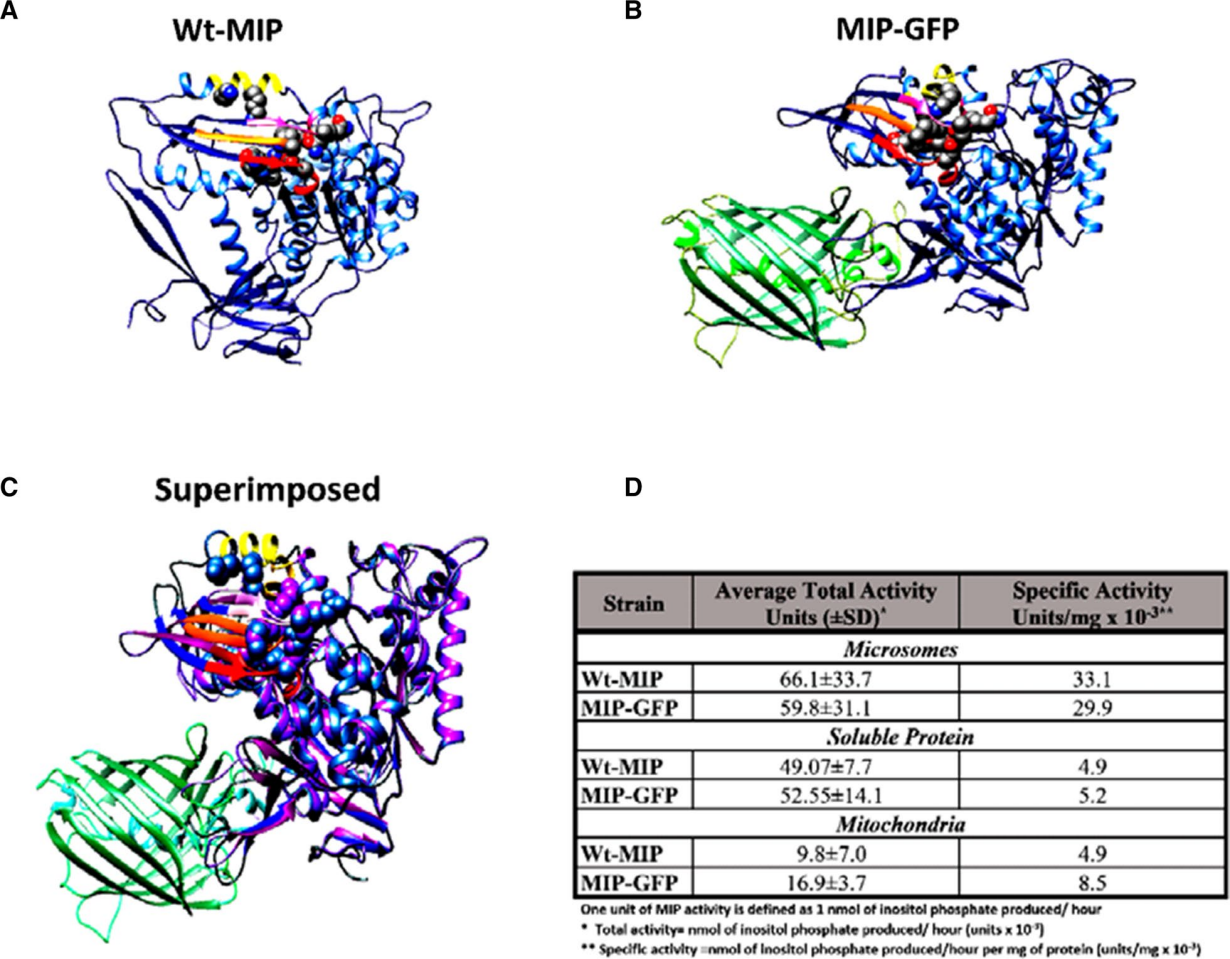
Comparative 3-D modeling of MIP-GFP and Wt-MIP proteins. 3-D protein models of Wt-MIP and MIP-GFP were created in PHYRE-2 [62]. Proteins are colored coded with Wt-MIP shown in (Blue) **A** MIP-GFP (Blue/Green) **B **Superimposed images [66] display Wt-MIP in (Blue) and MIP-GFP (Purple/Green), **C** MIP-GFP catalytic activity was compared to that of Wt-GFP, **D** Basak et al. [65] described six highly conserved amino acid patterns found in active sites of crystal structures from eukaryotes and prokaryotes. Four of the most highly conserved motifs are colored: HNVCEDSLL (Red), DSKVAMDEY (Orange), FRSKEISKS (Yellow), and YNHLGNNDG (Pink). Highly conserved amino acids, Ser323, Gly324, Gln325, Thr326, Lys369, Lys373, Lys412 and Lys489, are thought to be important for catalytic activity [65]. The local RMSD (root mean square deviation) for the two models, 1.61 Å, is < 2.0 Å, whereas the global RMSD, 3.65 Å, is > 2.0 Å. These RMSD values specify a conformational change with functional conservation [66]

To certify the use of a tagged enzyme in co-culturing experiments, microscopic, biochemical, and protein structural studies were employed to compare functional and conformational integrity of a GFP tagged MIP enzyme, MIP-GFP, (Table 1) with that of an untagged, wild-type MIP enzyme (Wt-MIP), (Table 1). Digital images obtained with a confocal microscope, equipped with DIC imaging capacity, showed that MIP-GFP fluorescence is associated with internal membranes, the cell wall, and the extracellular vesicles (Fig. 1). Likewise, detailed transmission electron microscopic (TEM) micrographs of Wt-MIP expression reflected confocal data, detecting Wt-MIP enzyme expression in equivalent cellular structures, including cell walls and extracellular vesicles (Fig. 2). Moreover, subsequent biochemical analyses, including protein purification, enzyme assays, and protein modeling, established functional and conformational integrity of MIP-GFP. Crystal structures and active site maps of a highly conserved enzyme allowed the creation of 3-dimensional (3-D) models of Wt-MIP and MIP-GFP, demonstrating conformational change with functional conservation (Fig. 3A–C). In addition, the local RMSD (root mean square deviation) for the two models, 1.61 Å.is less than 2.0 Å, indicating that the active site of the enzyme is not affected by the presence of GFP in MIP structure. In contrast,, a global RMSD of 3.65 Å is greater than 2.0 Å, reflecting structural differences between the models. This result is not surprising, given that Wt-MIP and MIP-GFP are expected to be dissimilar in the region where GFP sequence has been inserted into MIP sequence (Fig. 3A-3C). Enzyme activity assays and microscopic examinations showed that both MIP and GFP are functional in the fusion protein. Also, enzymatic activity detected in soluble and organelle proteins, supported equivalent subcellular locations identified in microscopic images (Fig. 3D).

**Table 1:** *S. cerevisiae* strains utilized in co-culturing experiments

Yeast strain	Genotype
SH 477 (Wild-Type) (Wt-MIP)*	(*MATα, URA3)*
INO1 Δ (Deleted INO1 Gene)*	*(MATα, URA3, INO1)*Δ
MIP:GFP(INO1:GFP fusion gene)	(*MATα,URA3, INO1:GFP*)

*Yeast strains were gifts from Dr. Susan Henry (Culbertson and Henry, 1975)

**Fig. 4:**
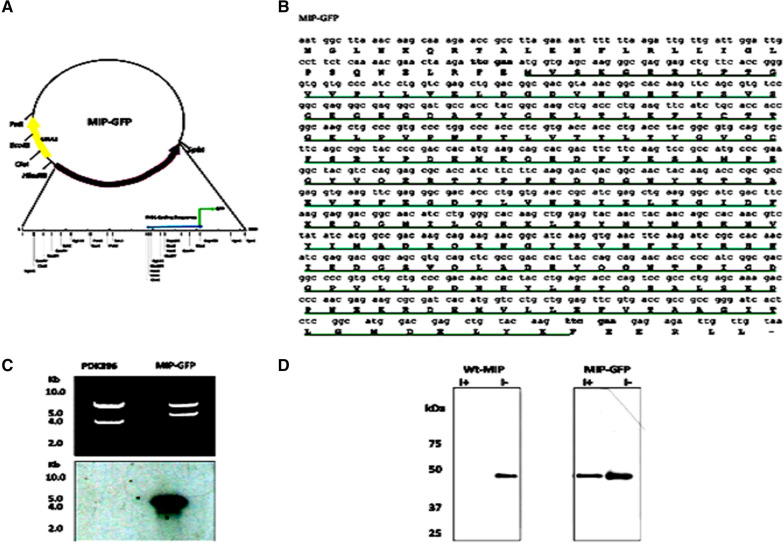
Construction of a MIP-GFP reporter enzyme. The complete INO1 gene, located on a 4.2 kb Hind lll- Kpn l restriction enzyme fragment, was cloned into a high copy number plasmid vector, pDK 396. **A** [51, 52], The plasmid was used to construct an INO1**:** GFP fusion gene, encoding a fluorescent MIP-GFP reporter enzyme. The INO1 gene encodes MIP in *S. cerevisiae* [51, 52]*.* **A** GFP gene [53], with Csp45I restriction site adapters, was inserted into a Csp45I restriction enzyme site (shown in bold type) located at the 3 prime end of INO1. DNA sequencing and bioinformatics analyses confirmed the correct reading frame**,** GFP is underlined. **B** while polymerase chain reaction cloning, using gene specific primers [54] and Southern blotting [55] confirmed the correct genomic location of the fusion gene after its integration into the yeast genome, using homologous recombination as described by Huh et al*.*[56], replacing resident genomic copy of the INO1 gene, (**C)**. Western blotting studies of MIP-GFP and WT-MIP organelles, found that MIP-GFP in the plasma membrane is less sensitive to inositol feedback inhibition than Wt-MIP (**D**)

Also, enzymatic activity detected in soluble and organelle proteins, supported equivalent subcellular locations identified in microscopic images (Fig. 3D). However, further characterization of organelles, employing western blotting, showed that MIP-GFP in the plasma membrane had a reduced response to regulation by exogenous inositol (Fig. 4D). Unlike Wt-MIP, MIP-GFP plasma membrane protein is not completely repressed when grown in the presence of exogenous inositol (Fig. 4D). It is known that cytoplasmic MIP is highly regulated by feedback inhibition of its end product, inositol, in *S. cerevisiae* (Fig. 4D). It appears that GFP inserted into the carboxyl, C-terminus, of MIP diminishes its robust response to exogenous inositol (Fig. 4D). This change may affect its movement into and out of plasma membranes without disturbing its catalytic activity, since all known active sires are preserved in the conformational change of MIP-GFP (Fig. 3A–C).

### Supernatant sources of inositol can sustain growth

**Fig. 5:**
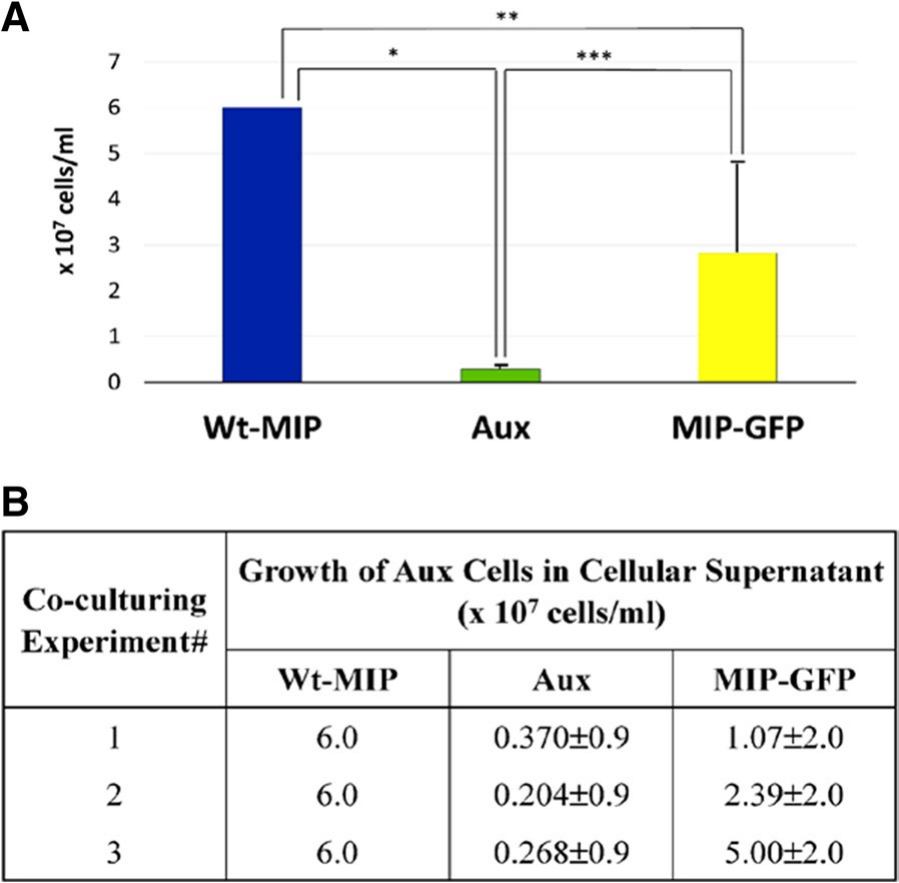
Growth of Aux cells in cellular supernatant. A bar graph, **A**, created from cell densities, **B**, illustrates and compares growth of Aux cells in three different transwell co-culturing experiments. Data documented sustained growth of Aux cells in Wt- MIP supernatant (positive control) (blue bar), no growth in Aux supernatant (negative control) (green bar), and mixed growth in supernatant from cells containing MIP-GFP reporter enzyme (yellow bar). ANOVA analyses and a plot of mean values show that there are significant differences in growth between the study samples (p < 0.05)

The idea that secreted MIP enzyme in the extracellular environment may function as a growth factor was challenged using a transwell co- culturing experimental approach [39–41]. A critical component of this method involved utilizing permeable membranes with extremely small pore sizes, 0.4 µm, in the upper chamber of the culture dish to prevent contamination or mixing of other cells with Aux cells. Since *S. cerevisiae* cells can vary greatly in size, anywhere from 4–10 µm, it was necessary to utilize 0.4 µm, the smallest pore size available. Densities of Aux cells, grown in the presence of supernatant from Wt-MIP, Aux, or MIP-GFP cells, were calculated after measuring the OD660 with a Genesys 2 spectrophotometer [42]. Cell densities for three different transwell co- culturing experiments are reported (Fig. 5). Statistical analyses and a plot of mean values show that there are significant differences in growth between the study samples (p < 0.05). A bar graph compares and illustrates these differences in cell densities (Fig. 5A). Blue bars denote sustained growth and efficient utilization of Wt-MIP supernatant as a source of inositol for Aux cells, while green bars indicate that supernatant from Aux cells cannot serve as an external source of inositol. Aux cells utilizing Aux cellular supernatant as a source of external inositol, failed to proliferate. Yellow bars illustrate limited growth of Aux cells consuming MIP-GFP supernatant as a source of inositol (Fig. 5). One plausible explanation for this occurrence is the finding that MIP-GFP has a reduced response to feedback inhibition by exogenous inositol in the plasma membrane, which may alter its ability to move efficiently into and out of plasma membranes, decreasing the amount of MIP-GFP protein present in the supernatant. Good growth in MIP-GFP supernatant requires efficient movement and concentration of extracellular vesicles in the intercellular environment, as with Wt-MIP. Given that mechanisms of transport and uptake are unknown and that all physical parameters are constant, the number of cells in each MIP-GFP growth experiment would depend on the number of vesicles free to move into and out of the supernatant. This number appears to slowly increase in MIP-GFP supernatant (Fig. 5).

### Microscopic examination of Aux cells grown in supernatant sources of inositol

**Fig. 6:**
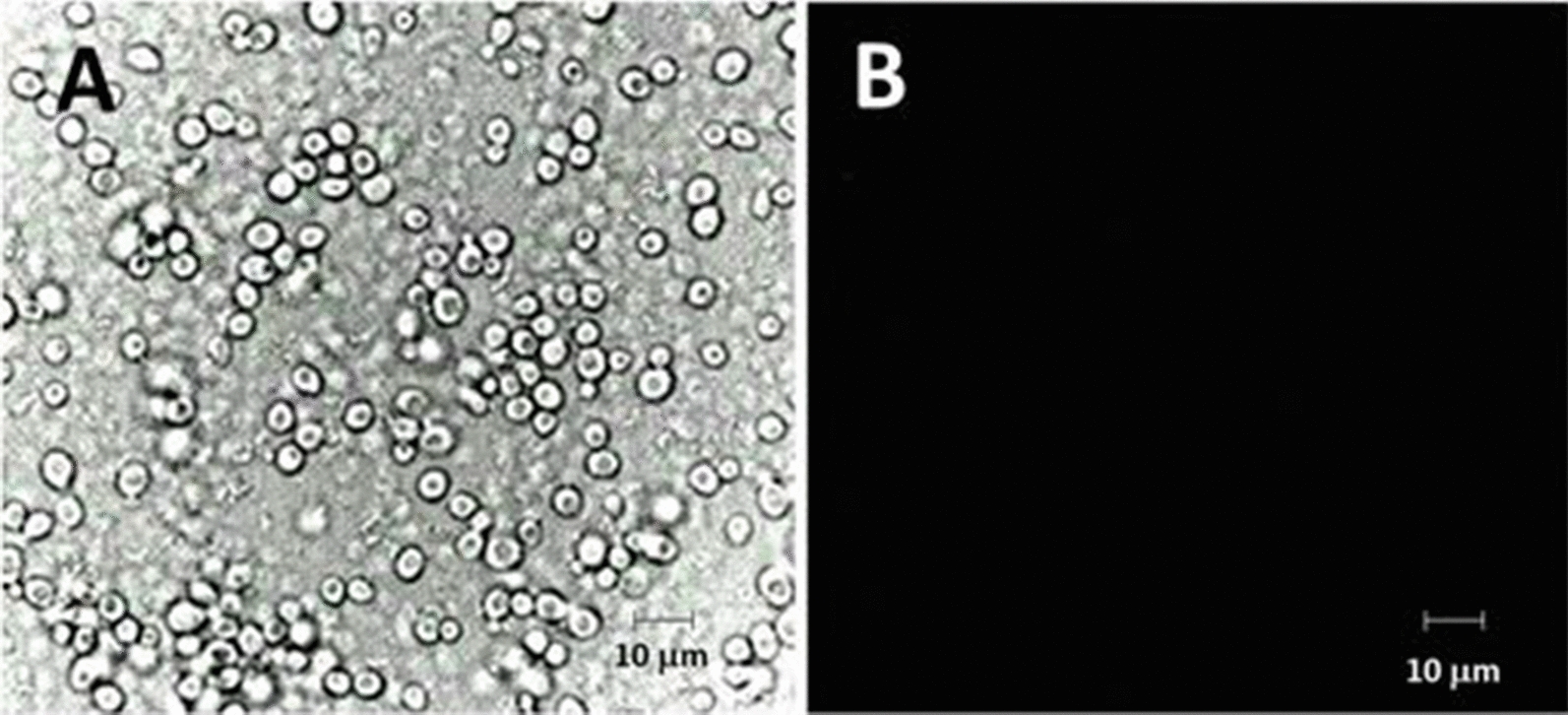
Growth of Aux cells utilizing Wt-MIP cellular supernatant as a [43] source of exogenous inositol. Micrographs, captured with visible light, show excellent growth of auxotrophic cells utilizing Wt-MIP cellular supernatant as an exogenous source of inositol 4A. Images taken with FITC wavelengths indicated that these cells contained no GFP or other contaminating fluorescent molecules 4B. Cells were examined for contamination by switching wavelengths and evaluating cells for continued glow under FITC, YFP, Cherry, and TRIC settings

**Fig. 7:**
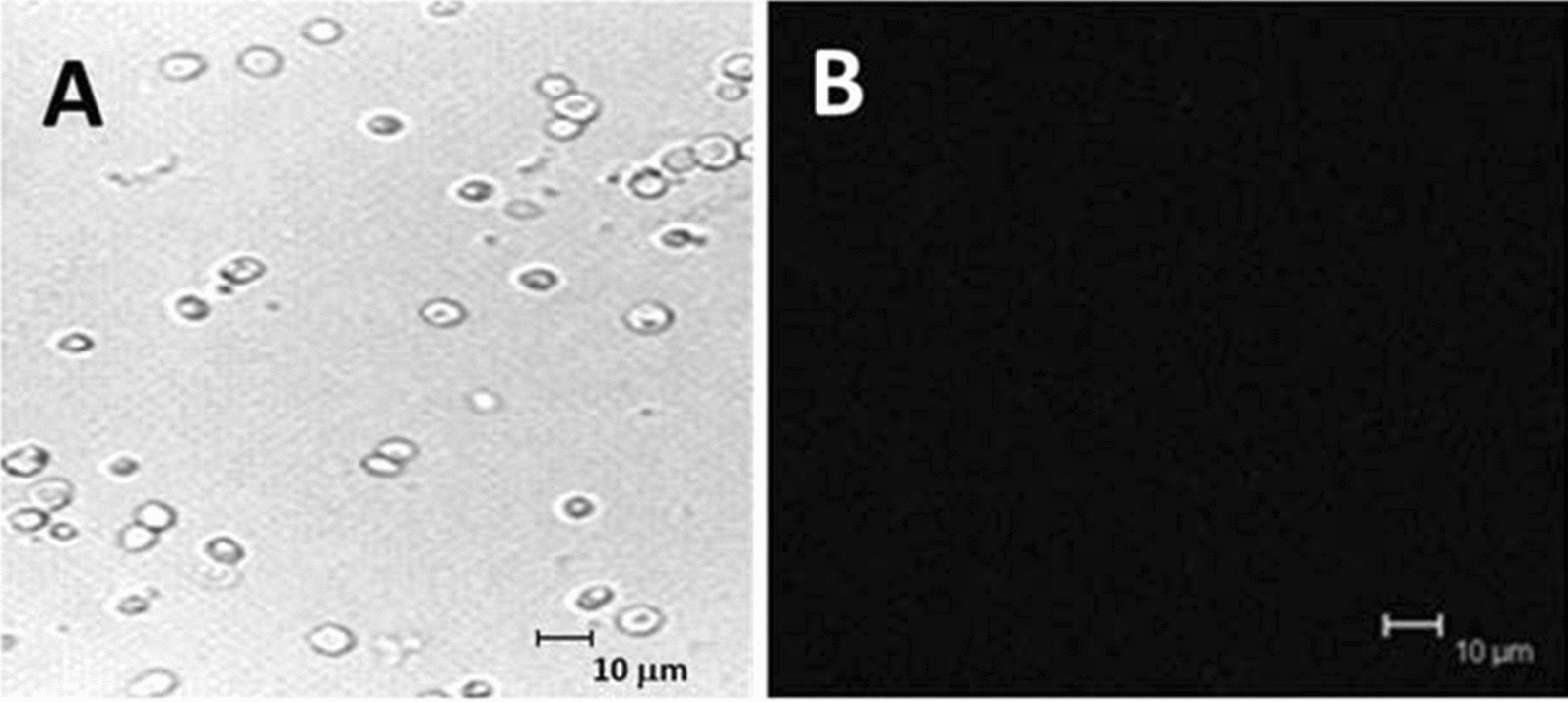
Growth of Aux cells in Aux cellular supernatant as a [43] source of inositol**. C**onfocal images, taken with visible light, showed that inositol requiring Aux cells cannot utilize auxotrophic cellular supernatant as a source of external inositol **A**. Low cell densities support this microscopic finding. Evaluating cells for continued glow under FITC, YFP, Cherry, and TRIC settings demonstrated a lack of contaminating fluorescent molecules **B**

**Fig. 8:**
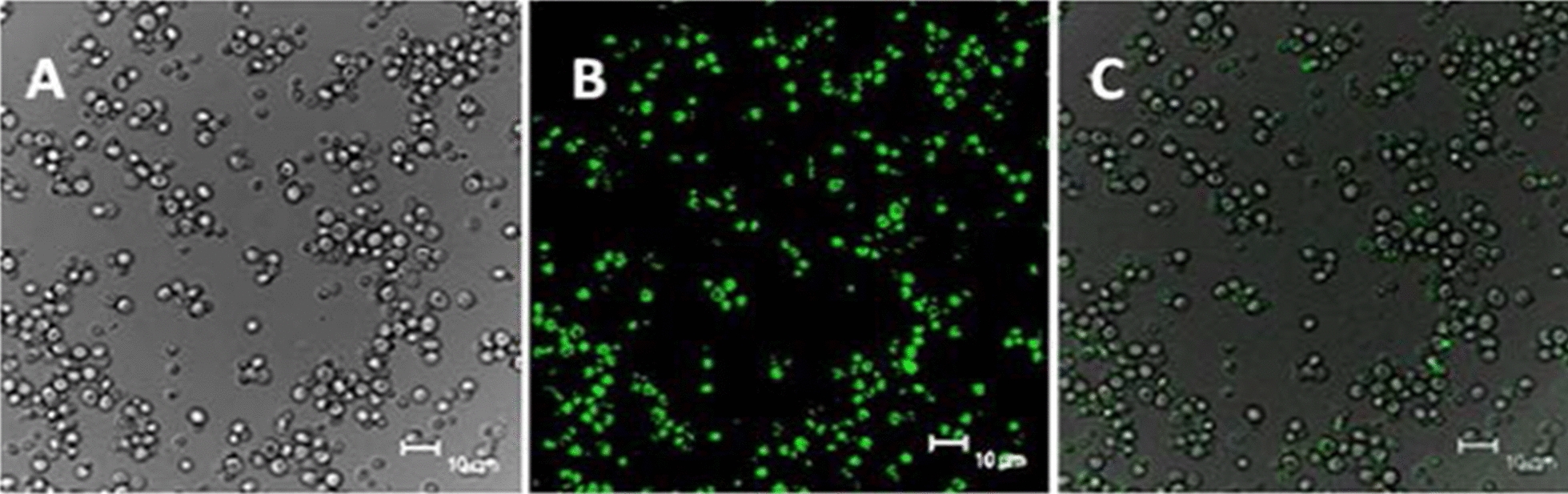
Growth of Aux cells in MIP-GFP cellular supernatant. Light micrographs show sustained growth of Aux cells cultured in MIP-GFP supernatant as a source of inositol **A.** Moreover, Aux cells identified in FITC mode revealed the uptake of fluorescent MIP-GFP molecules **B. V**isible and fluorescent images, **A** and **B,** respectively, were merged to show that MIP-GFP fluorescence aligns with **c**ells **C**

Given the results of transwell co-culturing experiments, utilizing cellular supernatants as external sources of inositol, and the fact that MIP has been detected in extracellular vesicles [15–17], it was imperative that we assess the fate of MIP-GFP secreted into the extracellular milieu of Aux cells. Toward this end, Aux cells were subjected to confocal studies, utilizing a confocal microscope with DIC imaging capacity, to visualize cells with both visible and fluorescent wavelengths (Figs. 6, 7, and 8).

Aux cells, located in the lower chamber of the culture dish, were removed and prepared as described then examined for MIP-GFP expression. Since GFP tagged molecules should only glow under FITC wavelengths, all cells were examined for contamination by switching wavelengths and evaluating cells for continued glow under YFD, Cherry, and TRIC settings as well. Common bacteria tend to give off fluorescence in a full spectrum of colors and are identified by this technique [43]. As shown in (Fig. 6), Aux cells grown in Wt-MIP supernatant were only visible with light (Fig. 6A), not fluorescent wavelengths (Fig. 6B), demonstrating that these cells did not contain MIP-GFP or any other contaminating fluorescent molecules. Moreover, cell densities indicated that Wt-MIP cellular supernatant provided an excellent, sustainable source of inositol for vigorous growth of Aux cells (5A). In contrast, supernatant from Aux cells could not sustain growth or function as a source of inositol (Fig. 7A). There was no proliferation of Aux cells, utilizing Aux supernatant. Additionally, cells weren’t visible with fluorescent wavelengths, confirming the absence of contaminating fluorescent molecules (Fig. 7B). Fluorescence was only detected in Aux cells grown in supernatant from cells containing MIP tagged with GFP, revealing the uptake of MIP-GFP (Fig. 8B). Corresponding light microscopic analyses confirmed that MIP-GFP supernatant provided a good source of inositol for sustained growth of *S. cerevisiae* inositol requiring Aux cells (Fig. 8A).

## Discussion

**Fig. 9:**
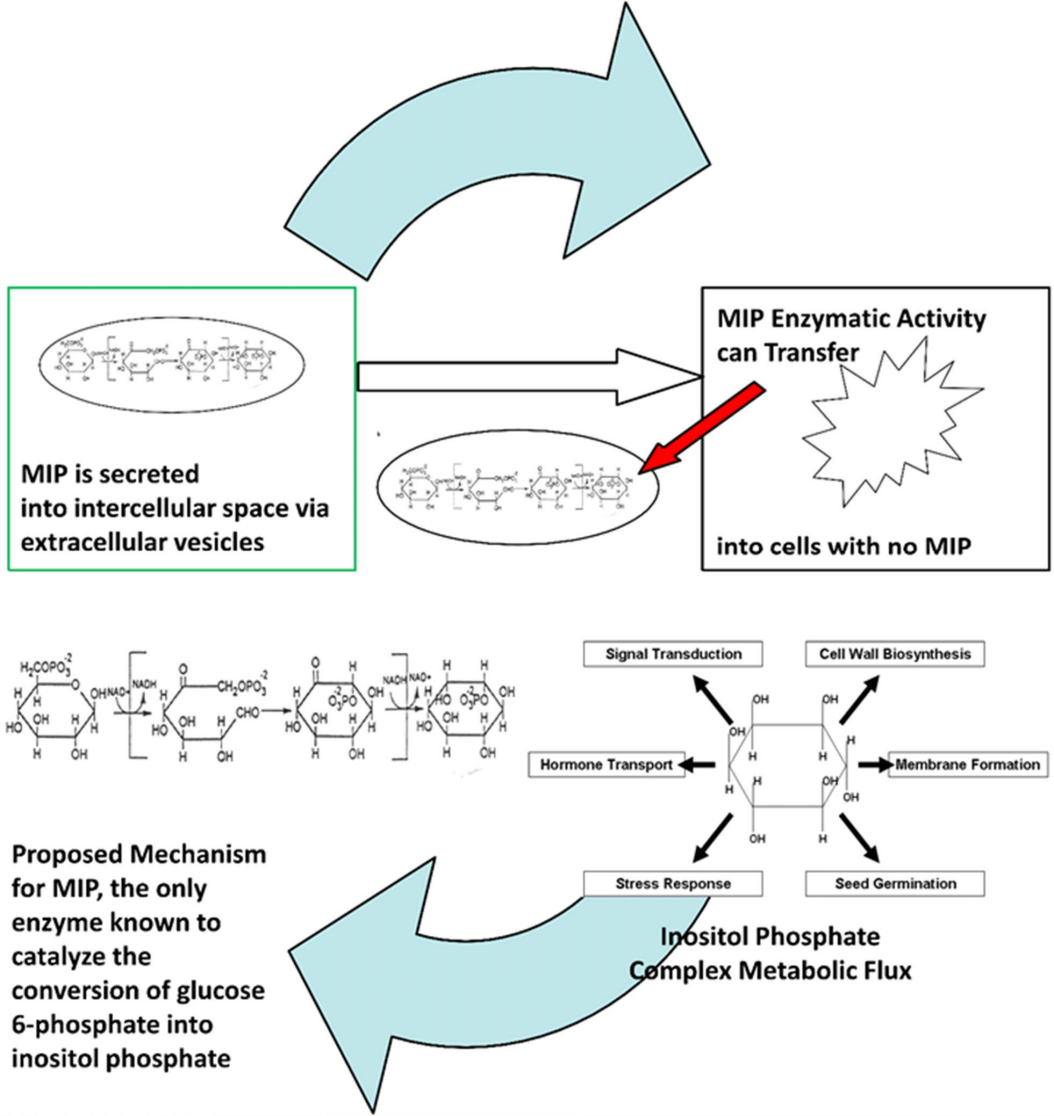
A diagram of inositol phosphate biosynthesis, its transport, and empirical functions. Inositol phosphate, the immediate precursor of free inositol, is generated from a tightly coupled oxidation and reduction reaction that requires NAD^+^ to proceed [6]. Proposed intermediates of this reaction, 5- ketoglucose 6-phosphate and inose, 2,1-phosphate, have not been isolated; it is believed that they remain tightly bound to the enzyme [6]

Movement of vesicles into and out of intercellular spaces is an evolutionarily conserved mode of cell–cell communication for diverse organisms, including prokaryotes and eukaryotes [26–28, 44]. Current studies made use of a well-studied model eukaryote, *S. cerevisiae*, to examine the fate of MIP secreted in extracellular vesicles. Consequently, transwell co-culturing experiments uncovered previously unknown functions for the enzyme. Data show that: (1) a *S. cerevisiae* inositol Aux cells can sustain growth in the presence of cellular supernatant sources of inositol, (2) this growth depends on the uptake of MIP, and (3) MIP can traverse cell walls. A schematic of these findings is detailed in (Fig. 9). In nature, some cells, such as *Schizosaccharomyces pombe*, are natural auxotrophs of inositol, yet depend on the nutrient for cell viability [45, 46]. Still other cells, with biosynthetic capacity to convert glucose 6-phosphate into inositol phosphate, may experience cellular events, which mutate or inactivate the catalytic activity of MIP. All things considered, the presence of inositol phosphate biosynthetic competency in the intra and intercellular milieu confers a selective advantage to all cells requiring inositol for growth and communication with other cells, especially those in multicellular organisms. Present research data suggest that MIP plays a vital role in regulating the complex metabolic flux of inositol phosphate. Moreover, cycling inositol phosphate biosynthesis from intracellular to extracellular environments ensures that an essential nutrient and cell–cell communicator is always available for its currently defined functions in all kingdoms of life, including central cell signaling pathways, membrane trafficking, stress response, autophagy, lipid metabolism, hormone transport, and neurodegenerative disorders [47, 48].Findings presented in this report have been demonstrated by others [49]. Published data show that fungal cells can bind and internalize extracellular vesicles [49]. This knowledge enhances their commercial potential, especially as it pertains to inositol production. A recent research article has demonstrated the complexity involved in engineering inositol production in bacterial cells [50]. Given the importance of inositol in many industries, including those related to health, pharmaceuticals, and agriculture, information presented here will allow a more efficient production of inositol and an extracellular vesicle in which to transport it.

## Conclusions

In summary, co-culturing experiments, designed to test a probable function for MIP secreted in extracellular vesicles, uncovered previously unknown functions for the enzyme. Present data suggest that MIP can: (1) move into and out of intercellular space, (2) traverse cell walls, and (3) act as a growth factor to promote cellular proliferation in an inositol requiring cell. These findings advance current knowledge concerning spatial control of inositol phosphate biosynthesis. Consequently, we can now employ new experimental approaches to define mechanisms that regulate the complex metabolic flux of inositol phosphate biosynthesis and delineate the role of an essential enzyme, capable of synthesizing an indispensable cell–cell communicator, in the process. Most importantly, resulting data identified a non-viral vector, i.e. an extracellular vesicle, capable of synthesizing and transporting inositol phosphate, a biological activity that can be harnessed to enhance and advance specificity of current inositol phosphate therapeutics.

## Methods

### *S cerevisiae* growth conditions

Yeast strains utilized in all experiments are listed in Table 1. Cells were grown at 30^0^ C in minimal medium (20 g glucose, 5.0 g ammonia acetate, 1.7 g yeast nitrogen base, 10 ml complete amino acid solution, 20 ml yeast vitamin solution per liter) with or without inositol or complete medium (20 g glucose, 20 g bactopeptone, 10 g yeast extract per liter).

### Construction and evaluation of a MIP-GFP reporter enzyme

The INO1 gene encodes the MIP enzyme in *S. cerevisiae* [51, 52]*.* Plasmid pDK396 [52], containing the complete INO1 gene located on a Hind lll- Kpn l restriction enzyme fragment, was used to construct an INO1**:** GFP fusion gene, encoding a fluorescent MIP-GFP reporter enzyme in *S. cerevisiae* cells. Specifically, a GFP gene [53], with Csp45I restriction site adapters, was inserted into a Csp45I restriction enzyme site located at the 3 prime end of INO1 (Fig. 4A). GFP is a 238 amino acid residue protein, which exhibits bright green fluorescence when exposed to light in the blue to ultraviolet range [53]. DNA sequencing and bioinformatics analyses confirmed the construct’s correct reading frame, while polymerase chain reaction cloning [54] and Southern blotting [55]confirmed the correct genomic location of the fusion gene after its integration into the yeast genome, using homologous recombination as described by Huh et al*.*[56], replacing the resident genomic copy of the INO1 gene (Fig. 4C). Western blotting of organelle proteins [15] further confirmed the insertion of GFP into the genomic copy of INO1 (Fig. 4D).

### MIP-GFP activity was assessed in soluble and membrane fractions of* S. cerevisiae*

#### Isolation of *S. cerevisiae* microsomes and mitochondria

Microsomes were isolated as described by Carmen and Fischl [58]. Yeast cells were grown overnight in yeast broth without inoitol. Microsomal fractions were further purified using a percoll density gradient [15]. Yeast mitochondria were isolated using a MITOISO3 kit (Sigma). Cells were grown overnight to a density of 1.3 (OD600), centrifuged at 3,000 g for 5 min, and washed with sterile water. We further purified the mitochondria using a percoll gradient. First 150 µl of percoll was added to each sample. resulting in a concentration of 15% percoll. Next, the samples to a were added to density gradient consisting of 2 ml of the following, 40% percoll and 23% percoll. Finally, the samples were centrifuged at 31,000 g for 15 min at 4 °C in a fixed angle rotor. Mitochondria were collected, diluted with 4 volumes of 1X storage buffer (Sigma), pelleted at 17,000 g for 15 min at 4 °C in a fixed angle rotor, concentrated in a Slide-A-Lyzer Mini Dialysis Unit (Pierce) and stored at − 20 °C. Isolated microsomes and mitochondria were solubilized overnight for purity and marker assays [15]**.**

#### Isolation of soluble proteins from *S. cerevisiae*

Soluble proteins were isolated as described by Lackey et al. [15] with modifications. Yeast cells were grown in 100 ml of media without inositol at 30 °C to an OD 600 of 1–1.5. Isolated soluble proteins were suspended in buffer containing a protease inhibitor cocktail (Roche).

#### Assay for MIP-GFP catalytic activity

MIP-GFP catalytic activity was evaluated according to Barnett et al*.*[57]**.** Soluble proteins (0.01 mg) and solubilized purified organelles (0.002 mg) were incubated as described. Phosphate concentrations, an indirect quantification of inositol phosphate, were measured in a Genesys 2 spectrophotometer at 820 nM and calculated using a standard phosphate curve (Fig. 3).

#### Confocal microscopic examination of MIP-GFP reporter enzyme

For confocal studies, *S. cerevisiae* cells, expressing MIP-GFP reporter enzyme, were grown overnight at 30 °C in minimal media without inositol. Wet mounts of cells were observed at 400X using a Leica Microsystems Heidelberg GmbH.

#### Preparation of cells for TEM studies of Wt-MIP expression

Cells of Wt-MIP strain SH 477 (Table 1), were grown to an OD660 of 1.0 in minimal medium without inositol supplementation and fixed for electron microscopy as described by Wright [59] with the following modifications. A 1:1 solution of yeast cell culture and fixative (0.2 M PIPES [pH 6.8], 0.2 M sorbitol, 2 mM MgCl2, 0.2 mM CaCl2, 0.5% glutaraldehyde, 4% formaldehyde) was incubated at room temperature for 5 min, centrifuged, suspended in fixative,diluted 1:1 in sterile water. Cells were collected, suspended in 25 ml of sterile water, incubated for 10 min, and pelleted at 1,500 RPM. Finally, after 2X washes in 25 ml of sterile water, cells were suspended in 5 ml of 1% sodium metaperiodate at room temperature for 15 min. Following incubation, cells were collected at 1500 RPM, washed with 5 ml of sterile water, suspended in 5 ml of 50 mM ammonium chloride, and incubated 15 min at room temperature.

Afterwards, cells were collected, washed 2X in sterile water, and collected via centrifugation. Following collection, cells were dehydrated in a graded series of ethanol from 60 to 100% for 10 min each, followed by infiltration and embedding [59]**,** using London Resin (LR) Gold instead of LR-White. Polymerization of the resin was carried out by exposure to UV light for 48 h. Thin sections were prepared and stained with uranyl acetate and lead citrate as described b y W r i g h t [59]. Stained grids were submerged in 20 ml of TBSTO (140 mM NaCl, 3 mM KCl, 8 mM, Na2HPO4, 1.5 mM KH2PO4, 0.05% Tween-20, and 2% ovalbumin), transferred to yeast MIP antibody, diluted 1:1000 in TBSTO, and incubated for 2 h at room temperature in a moist chamber. Afterwards, grids were washed 3X in TBST (140 mM NaCl, 3 mM KCl, 8 mM, Na2HPO4, 1.5 mM KH2PO4, 0.05% Tween-20), submerged in 20 ml of TBSTO, incubated for 15 min, transferred to gold-conjugated secondary antiserum, diluted 1:100 in TBSTO, washed 3X in TBST, fixed with 5% glutaraldehyde in TBS (140 mM NaCl, 3 mM KCl, 8 mM, Na2HPO4, 1.5 mM KH2PO4) for 1 h, washed 3X in TBST, followed by 3X washes in TBS and water, air dried, stained with uranyl acetate and lead citrate for TEM analyses.

#### Transwell co-culturing experiments

Co-culturing experiments utilized Corning™ transwell cell culture plates with twelve wells, six wells in an upper chamber of the plate with six corresponding wells in a lower chamber. Wells in the upper chamber contained polyester permeable membranes, 24 mm in diameter, 10 µm thick, with 0.4 µm pore sizes. To prepare inositol auxotrophic, Aux, cells for the lower chamber, cells were grown overnight at 30 °C in minimal medium with inositol to prevent inositol-less death [60], centrifuged, washed 2X in minimal medium without inositol, and diluted in minimal medium without inositol to a final concentration of 0.128 × 10^7^ cells/ml. To co- culture these cells with three different supernatant sources of external inositol, 1 ml of Aux cells were placed in each of six wells in the lower chamber of the culture dish. Cellular supernatant was obtained from Wt-MIP and MIP-GFP cells grown in minimal medium without inositol, while Aux cells were grown in complete medium, which has a minimal amount of inositol. After growing cells to an OD660 of 1.0 (1.85 × 10^7^ cells per ml), supernatant was isolated via centrifugation and sterilized, using a 0.2 µm Nalgene rapid-flow sterile filter. Sterile supernatant (1 ml), containing either Wt-MIP proteins, Aux, no MIP proteins, or MIP-GFP reporter proteins, was placed in the upper chamber in wells containing permeable membranes with an extremely small pore size, 0.4 µm. Aux cells in the lower chamber of the culture plate were removed after incubation at 30 °C for 24 h in the presence of different cellular supernatants as sources of external inositol, quantitated using a Genesys 2 spectrophotometer, and examined for MIP expression using confocal microscopic analyses. Three co-culturing experiments were performed, using isolated supernatants.

#### Imaging Aux cells grown in diverse supernatant sources of external inositol

A Zeiss Axioscope 40 confocal microscope with differential interference contrast (DIC) imaging capacity was used to visualize yeast cells with both visible and UV wavelengths. One ml of auxotrophic cells, located in the lower chamber of the culture dish, was removed, centrifuged, washed 2X in minimal medium with no inositol, and evaluated for MIP expression. Fluorescence was observed with wavelengths from 395 to 580 nm. Cells were examined for contamination by switching wavelengths and evaluating cells for continued glow under FITC, YFP, Cherry, and TRIC settings. Common bacteria tend to give off fluorescence in a full spectrum of colors and are identified by this technique [43].

#### Assessing structural and functional conservation of MIP-GFP

Protein models of Wt-MIP and MIP-GFP proteins were generated in SWISS-MODEL [61], PHYRE-2 [62] and I-TASSER [63]. All models were compared to crystal structures of MIP, located at the RCSB Protein Database (www.rcsb.org). Sequence homology and secondary structure were examined in Chimera 1.14 [64]. In all models generated by SWISS-MODEL, MIP and GFP were two separate 3D structures. Further examination of the protein sequence in Chimera 1.14 showed that the program removed GFP protein sequence from MIP protein sequence. Models generated by I-TASSER accurately generated GFP but not MIP. Examination of MIP active sites, predicted by I-TASSER, showed non-conserved amino acids, as defined by site directed mutagenesis [65, 67] Models examined in Chimera 1.14 showed errors in MIP secondary structure based on comparison to MIP crystal structures. Only models produced by PHYRE-2 were selected because this software was able to extract a MIP-GFP fusion protein. In addition to similarity to known protein sequences, PHYRE2 used protein structure prediction to generate 3D protein structures. Superimposed models and RMSDs]were produced using SuperPose [66]. Models shown in (Fig. 3) were colored in Chimera 1.14. [64].

## Data Availability

Tools and materials used to generate data from this research, antibody and yeast strains, were gifts as acknowledged in acknowledgements.
